# Using Pop-GUIDE to Assess the Applicability of MCnest for Relative Risk of Pesticides to Hummingbirds

**DOI:** 10.3390/ecologies4010013

**Published:** 2023-03-03

**Authors:** Matthew A. Etterson, Elizabeth A. Paulukonis, S. Thomas Purucker

**Affiliations:** 1 US Environmental Protection Agency, Office of Research and Development, Center for Computational Toxicology and Exposure, 6201 Congdon Blvd, Duluth, MN 55804, USA; 2 Oak Ridge Institute for Science and Education, 109 TW Alexander Dr, Durham, NC 27709, USA; 3 US Environmental Protection Agency, Office of Research and Development, Center for Computational Toxicology and Exposure, 109 TW Alexander Dr, Durham, NC 27709, USA

**Keywords:** MCnest, Trochilidae, Pop-GUIDE, avian pollinator, hummingbirds

## Abstract

Hummingbirds are charismatic fauna that provide important pollination services, including in the continental US, where 15 species regularly breed. Compared to other birds in North America, hummingbirds (family Trochilidae) have a unique exposure route to pesticides because they forage on nectar. Therefore, hummingbirds may be exposed to systemic pesticides borne in nectar. They also may be particularly vulnerable to pesticide exposure due to their small size and extreme metabolic demands. We review relevant factors including hummingbird life history, nectar residue uptake, and avian bioenergetic considerations with the goal of clearly identifying and articulating the specific modeling challenges that must be overcome to develop and/or adapt existing modeling approaches. To help evaluate these factors, we developed a dataset for ruby-throated hummingbirds (*Archilochus colubris*) and other avian species potentially exposed to pesticides. We used the systemic neonicotinoid pesticide imidacloprid as an illustration and compared results to five other common current use pesticides. We use the structure of Pop-GUIDE to provide a conceptual modeling framework for implementation of MCnest and to compile parameter values and relevant algorithms to predict the effects of pesticide exposure on avian pollinators. Conservative screening assessments suggest the potential for adverse effects from imidacloprid, as do more refined assessments, though many important limitations and uncertainties remain. Our review found many areas in which current USEPA avian models must be improved in order to conduct a full higher-tier risk assessment for avian pollinators exposed to neonicotinoid insecticides, including addition of models suitable for soil and seed treatments within the MCnest environment, ability to include empirical residue data in both nectar and invertebrates rather than relying on existing nomograms, expansion of MCnest to a full annual cycle, and increased representation of spatial heterogeneity. Although this work focuses on hummingbirds, the methods and recommendations may apply more widely to other vertebrate pollinators.

## 1. Introduction

Avian pollinators contribute vital ecosystem services and are a key part of global food security, but anthropogenic stressors threaten many of these populations [1–3]. Hummingbirds (Aves; Apodiformes; Trochilidae) are charismatic avian pollinators that have unique physiological and life history traits that distinguish them from other avian species. Unlike most birds, hummingbirds rely on plant nectar to sustain their high metabolism. In North America, populations of hummingbirds have declined overall since the 1970s [4]. Previous studies have examined drivers of declines such as habitat loss or climate change for some species [5,6]; however, although field studies have detected pesticides in the tissues and cloacal fluid of hummingbirds [7–9], little work has focused on pesticide exposures. As pollinators, hummingbirds are susceptible to pesticides found in anthropogenic landscapes, yet little is known regarding the mechanisms of exposure and subsequent effects or how these effects may impact reproductive life cycles. Although some work has sought to quantify the presence of neonicotinoid pesticides in wild populations [7], no studies have focused on development of a quantitative model for estimating reproductive impacts from potential exposures.

Hummingbirds are endemic to the Americas and are one of three families, together with the treeswifts (Hemiprocnidae) and swifts (Apodidae), in the order Apodiformes. The International Ornithological Committee recognizes 361 species of hummingbirds in 113 genera [10]. Peak diversity in the family occurs in South and Central America, and around 47 species regularly occur in the United States and associated territories [11], 15 of which breed in the continental United States [10]. Following their split from their common ancestor with the swifts ~40–45 MYA, hummingbirds have evolved into highly specialized nectar consumers, though invertebrate prey remain an important component of their diet, especially during breeding [11]. Hummingbirds are known to provide pollination services to over 7000 flowering plants, with which they have extensively coevolved during successive adaptive radiations [12]. The rapid North American radiation of the bee hummingbirds (Mellisugini) is relatively recent, beginning 6–7 MYA [12,13].

Hummingbirds are among the smallest birds: US species range from 2.5 to 8.4 g. They can forage on thousands of flowers per day and have evolved numerous specialized traits associated with nectar consumption. These include an elongated bill and a bilobed semi-tubular tongue for probing flowers and extracting nectar [11]. Hummingbirds have proportionately large keels and specialized wing anatomy that permits hovering at flowers, an energetically costly activity. While hovering, hummingbirds may beat their wings up to 80 times/s, their heart rate may exceed 20 beats/s, and their respiration rates may exceed 4 breaths/s [11]. Their metabolic rates are the highest known for any vertebrate [14]. These high energetic demands are offset by high food consumption rates for their body size. For example, captive Anna’s hummingbirds (*Calypte anna*, 4.6 g) were estimated to require 9.8 g of nectar/day to meet daily energy needs [15]. Most hummingbirds also regularly consume insects [11]. Hummingbirds are highly altricial, hatching with eyes closed and without feathers, and require an extended period of post-hatching care as they develop toward fledging [11]. Clutch sizes are also small, typically only two eggs.

Like many insect pollinators exposed to pesticides [16], similarities in foraging habits [17] suggest that hummingbirds may be exposed to pesticides via nectar. Although more recently developed insecticides are designed to be less toxic to vertebrates than to insects, the high energetic demand of hummingbirds, resulting in exceptionally high food consumption rates, could expose them to higher levels of pesticides than other vertebrates. Conversely, high metabolic rates in hummingbirds may result in fast elimination rates (e.g., [18]), potentially reducing adverse effects of exposure. These potentially conflicting associations between exposures and effects make it difficult to predict whether hummingbirds are likely to be more or less vulnerable to pesticides than other birds. In addition, avian reproduction tests are typically performed on much larger birds, for example, northern bobwhite (*Colinus virginianus*, 178 g) or mallard (*Anas platyrhynchos*, 1082 g) compared to hummingbirds, though acute testing requirements now include a passerine study.

Field studies have implicated juvenile recruitment (survival of juveniles until first breeding) as a potential cause of long-term decline in the rufous hummingbird, *Selasphorus rufus*, [19]), a result supported by modeling results demonstrating that fitness in short-lived birds that reach sexual maturity after one year is necessarily most sensitive to changes in first-year survivorship [20]. Long-distance migration is relatively rare in the Trochilidae: of the 361 species, less than 10% (29) are known to perform long-distance latitudinal migrations, but this includes many of the temperate breeding US species [18,21]. These migrations likely contribute significantly to the survival and recruitment challenges faced by temperate hummingbirds as they do for other species with similar life cycles [22]. Of the eight most abundant US hummingbirds, four species are experiencing population declines [3]. These considerations, together with the timing of pesticide use and exposure that typically overlaps with nesting season, argue for an integrated approach to assessment that accounts for reproduction and early life survival.

Systemic insecticides, a class of insecticides that are absorbed by and persist in plant bodies and include neonicotinoids, are widely used in crop landscapes and have become a highly popular mode of pest control [23,24]. Hummingbird diets consist primarily of nectar and insects and therefore raise concern that pesticides, particularly systemic insecticides, may pose a twofold threat because of dual exposures and higher overall food consumption rates. Residues in pollen and nectar can vary widely depending on crop, timing, application type, and formulation. Predicted doses, based on pollen and nectar residues, often exceed the median lethal dose (LD50) for various invertebrate pollinators [25]. Several studies have documented measurable traces of neonicotinoids in hummingbirds studied in crop landscapes in western Canada [7], and recent work has provided the first look at targeted neonicotinoid exposure and subsequent metabolic effects [18].

In this manuscript we evaluate the Markov Chain Nest Productivity (MCnest, [26,27]) model and its associated exposure and effects models for their applicability to hummingbirds exposed to pesticides through consumption of both nectar and invertebrates. We consider the unique ecology and physiology of hummingbirds with a special focus on characteristics that make them vulnerable to pesticide exposure and effects compared to other birds. We develop a worked example to estimate fecundity impacts for hummingbirds and other selected avian species for comparison purposes and conclude with recommendations for modifications to existing methods to increase their suitability for hummingbirds and other avian pollinators. Our review seeks to answer the following questions. (1) Can hummingbird exposure and effects be modeled using existing risk assessment tools in the USEPA avian risk assessment toolbox? (2) What enhancements to the current toolbox would make hummingbird risk assessment more realistic and more accurate? (3) What risk predictions do current tools make about hummingbird risk and how does this compare to other species and between pesticide classes? We provide an illustration of hummingbird risk assessment procedure using existing EPA tools, but our analysis is not a complete risk assessment.

## 2. Materials and Methods

We use the Pop-GUIDE framework [28] to review available models, data, and literature on pesticide toxicity, exposure modeling, pesticide residues in nectar, and hummingbird life history. Pop-GUIDE is a multiphase process flow that we use to structure our review and document the relevant data and methods for assessing pesticide risk to hummingbirds. We also use Pop-GUIDE to document methods currently lacking that prevent USEPA from conducting higher-tier risk assessments for avian pollinators. The Pop-GUIDE process includes phases for defining model objectives, compiling data, making decisions about what processes to include in the model, and integrating these decisions into a conceptual model. We consider potential pesticide exposures and present data and equations for predicting hummingbird daily energetics, foraging, and exposures during the breeding season to estimate impacts on fecundity. We identify methodological gaps and point to areas requiring further research. The final phase of Pop-GUIDE is implementation and evaluation of the selected computational tools and methods. A major finding is that several important methods are lacking (reviewed below), and thus we do not conduct a risk assessment. Instead, we use MCnest to model a spray application of imidacloprid and provide an example of how hummingbird risk assessment could be implemented.

Refined, or higher-tier, risk assessment for birds at USEPA is done using the MCnest model [26,27]. MCnest is a model of the avian breeding season that simulates the progression of a nesting attempt from pair formation through a series of typical phases until nestling birds fledge from the nest, at which point females can renest or not according to typical species-specific propensities [29]. The model incorporates test endpoints from three standard avian toxicity tests submitted to USEPA by chemical registrants. The Avian Acute Oral Toxicity test [30] gives an estimate of the dietary dose of a pesticide that is estimated to be lethal to 50% of tested adult birds (LD50). The Avian Dietary Toxicity Test [31] gives an estimate of the dietary concentration expected to be lethal to 50% of tested juvenile birds (LC50). The Avian Reproduction Test [32] gives estimates of the highest dietary concentrations to breeding birds at which no effects are observed across a suite of reproductive endpoints, such as eggshell thinning, weight loss among breeding birds, egg viability, and post-hatching survival of chicks (collectively the no observed adverse effects concentrations, NOAECs). Due in large part to husbandry challenges, reproduction tests are typically conducted using landfowl (Galliformes, e.g., northern bobwhite, *Colinus virginianus*) or waterfowl (Anseriformes, e.g., mallard, *Anas platyrhynchos*). Current acute (LD50) testing requirements now include a passerine bird, most of which are much closer in body weight to hummingbirds. MCnest incorporates two exposure models, the Terrestrial Residue Exposure Model (T-REX, [33]) and the Terrestrial Investigation Model (TIM, [34]). T-REX is a general, conservative, lower tier model used for screening level assessments. TIM is a refined model that incorporates energetics and toxicokinetics for a more refined understanding of risk when indicated by screening assessments. Much more information on MCnest, T-REX, and TIM is provided below.

To illustrate methods currently available for higher-tier risk assessment for hummingbirds we develop a life history profile for ruby-throated hummingbird (*Archilochus colubris*; hereafter RTHU), the only hummingbird species to regularly breed in the eastern continental USA. We also develop a parameter set for an example pesticide applied to a representative crop. For this effort, imidacloprid was chosen as an example because it is a commonly used neonicotinoid insecticide, one to which hummingbirds may be exposed [7] and is considered highly toxic to birds [35]. Soybean was chosen to represent a widely planted crop on which imidacloprid and other pesticides are used. Using the RTHU profile, we simulate spray applications of pesticides on soybeans using MCnest. The chosen scenario may represent one in which risk appears low; the most recent screening level usage analysis (SLUA) conducted by US EPA estimates that approximately 50,000 lbs active ingredient of imidacloprid is used on less than 2.5% of soybean crops in the United States [36] annually, making the scenario we model a relatively rare one. Further, we could find no direct evidence that RTHU feed on soybean nectar. However, they are known to forage on other species of Fabaceae. Also, hummingbirds are known pollinators of blueberry crops in Argentina [37], suggesting they will forage in crops in some circumstances. For the example presented herein, we are primarily interested in exposure through spray drift as birds forage on preferred nectar sources adjacent to treated fields and we compare the conservative assumption that birds do forage on soybean to a more realistic assumption that they only forage in adjacent habitats off the treated field.

MCnest modeling involved two comparisons. First, MCnest predictions for RTHU exposed to imidacloprid in the soybean scenario were compared to other avian species to evaluate the relative sensitivity of hummingbirds under similar application conditions. Second, for just RTHU, imidacloprid was compared to other commonly used insecticides to give a measure of relative risk among insecticides to RTHU. The latter comparisons also used previously published toxicity data on five insecticides [38]. Current use information and labels for soybean applications for imidacloprid, λ-cyhalothrin, indoxacarb, and permethrin were taken from the USEPA Pesticide and Product Label System (https://ordspub.epa.gov/ords/pesticides/f?p=PPLS:1, accessed on 6 July 2022). Application rates for chlorpyrifos (for which registered uses of chlorpyrifos on food crops are subject to cancellation under the Federal Insecticide, Fungicide, and Rodenticide Act) and methomyl were taken from recently published Endangered Species Biological Evaluations [39,40]. Modeling for all pesticides included dietary exposure through consumption of both nectar and invertebrates.

## 3. Results

We structure our results and discussion of hummingbird exposure and effects by sequentially addressing the five phases of the Pop-GUIDE process. Throughout imidacloprid and RTHU are used as examples. Comments about the adequacy of MCnest and associated exposure models for the specific insecticide-species pair can often be interpreted to pertain more broadly to refined risk assessment for insecticides and hummingbirds in general, as will be clear from the context.

### 3.1. Identifying Model Objectives (Phase 1)

The first phase of the Pop-GUIDE process concerns identifying model objectives and uses the applicable regulatory risk assessment process to evaluate the trade-offs needed to achieve the required level of realism and precision from the model output. An important determinant of modeling objectives is the regulatory context under which the models will be developed and/or deployed.

The USEPA registers pesticides under the Federal Insecticide, Fungicide, Rodenticide Act (FIFRA) with, among other standards, a goal of not causing unreasonable adverse effects on the environment (40 CFR Parts 155, 158). Conceptually, the USEPA ecological risk assessment methodology for pesticides is tiered, with an increasingly rigorous set of testing requirements that can be combined with open literature data to evaluate hazard and risk. Tiered testing requirements for insect pollinators are focused on honey-bees [41–45] but include methods for modifying testing and estimating concentrations and persistence of residues on plants to account for pollen and nectar [39,42–44]. Screening level assessments for birds use Risk Quotients (RQ = exposure/effect), which compare modeled exposure predictions (numerator) to effects (denominator) measured in standardized toxicity tests [30,31,45]. When RQs suggest the potential for adverse effects, USEPA may escalate tiers to further investigate risk using MCnest to assess effects on mortality and fecundity. The T-REX and TIM exposure modules in MCnest provide a progression from general screening level (T-REX) to more refined (TIM) exposure assessments. T-REX takes a general approach, estimating exposure via food consumption, which is assumed to depend on body weight and dietary categories. TIM also uses dietary categories, but combines them with bioenergetics and toxicokinetics to estimate consumption and exposure, as may be required to capture the unique bioenergetics of hummingbirds. The review below centers on the applicability and limitations of these existing models in the context of higher-tier (beyond RQ) risk assessment for hummingbirds.

A primary output of the first phase of Pop-GUIDE is classifying the modeling objectives within the Levins [46] trichotomy that one typically optimizes generality, realism, or precision (though more recent interpretations have questioned whether these tradeoffs are inherent and unavoidable [47,48]). Given our focus on hummingbirds, the issue of generality at the taxon level motivates our review. The MCnest model is designed to give refined assessments of risks at the avian level by allowing it to be parameterized with species- and chemical-specific parameters. However, many algorithms and extrapolation methods commonly used in ecological risk assessment (and incorporated into MCnest) are more broadly fit at taxonomic levels of class or higher. Therefore, we evaluate methods and parameter alternatives by increasingly making modeling assumptions more specific to hummingbirds (sacrificing generality) so we can increase realism and precision with respect to predicted ecological risk metrics, such as fecundity, survivorship, individual fitness, and population status following chemical exposures. Our approach is motivated by some of the exceptional physiological characteristics of hummingbirds and food consumption behaviors relative to other birds (and other vertebrates in general).

In summary, the objectives for hummingbird higher-tier risk assessment must: (i) be general enough to apply to any US hummingbird species (and ideally any other avian pollinator); (ii) be specific enough to account for the unique biology and physiology of hummingbirds; (iii) account for the diverse application methods used for pesticides to which hummingbirds might be exposed (e.g., foliar spray, seed treatments, soil treatments); (iv) account for fate and transport processes governing the pesticide in the environment; and (v) account for all routes of exposure to which hummingbirds may be susceptible.

### 3.2. Compiling Available Data (Phase 2)

The second phase of the Pop-GUIDE process consists of compiling available data that will be used to parameterize the modeling effort. This includes relevant biological, chemical, and environmental data. The Pop-GUIDE structure describes data inputs for population models, which MCnest does not. It predicts effects on specific population endpoints (e.g., survival and fecundity) that would, in turn, be used in a population model. Therefore, data inputs were compiled to represent relevant parameters in the MCnest framework.

MCnest has a standard species library [49] consisting of 59 life history profiles for US birds known to use agroecosystems, none of which are pollinators. For this work we developed and added a profile for RTHU (Table 1), taking data from the authoritative Birds of the World account [50]. Because RTHU is an eastern North American species and all other US hummingbirds (excluding Caribbean species) are western, the use of RTHU is an incomplete representation of North American hummingbirds, and suggests a need to also develop profiles for some western species. For a very refined assessment, such as a spatially explicit population model using MCnest fecundity predictions [51], additional information on survival, migration, and spatial heterogeneity would be needed (reviewed in more detail below).

Avian toxicity data for MCnest modeling is available in three toxicity studies submitted to USEPA by chemical registrants, the avian reproduction test, the dietary toxicity test, and the acute toxicity test [30–32], with example data for imidacloprid provided in Table 2. Data on chemical properties for modeling fate and transport (e.g., chemical half-life, residues on potential dietary items, volatility) are also available from additional registrant submitted studies and in the general scientific literature. Example data for imidacloprid is given in the Supplementary Material. Pesticide usage in North America is governed by USEPA approved product labels, which are publicly available in the Pesticide Product and Labeling System (PPLS) database (https://ordspub.epa.gov/ords/pesticides/f?p=PPLS:1, accessed on 6 July 2022). Labels are often crop- and region-specific and may have modifications and/or exclusions within the range of federally listed species.

### 3.3. Identifying Model Algorithms (Phase 3)

The third phase of Pop-GUIDE is to identify the main algorithms that will be used by the exposure and effects modules in order to integrate these routines with larger environmental and population-level processes. These are broken down as a series of steps that include life history, growth and reproduction processes, relevant spatial and temporal population factors, and other factors that can range from diet to inter-species interactions. As noted previously, the MCnest model describes effects to specific population endpoints; therefore, we discuss the algorithms relevant to the MCnest framework and identify factors of importance.

#### 3.3.1. Exposure

Hummingbird exposure to pesticides is a function of numerous processes, including release of the chemical to the environment, fate and transport of the chemical following release, and exposure to the chemical (e.g., through diet, inhalation, or dermal exposure). These factors are reviewed below considering existing USEPA modeling methods for pesticides.

Methods to estimate media concentrations and received dose on or near pesticide-treated fields are a function of the application type, with three application types relevant to hummingbirds: foliar spray, seed, and soil treatment. Most pesticide exposure for hummingbirds, regardless of application method, is expected to be through ingestion of contaminated food (nectar and invertebrates). Other relevant exposure routes may include water ingestion, preening, inhalation, and indirect (dermal) exposures from leaf and tree surfaces. For foliar and seed treatment events, acute exposures from direct aerial contact and inhalation are also possible. T-REX and TIM differ considerably in intended use, complexity, and methods for representing components of exposure, with T-REX predicting only dietary exposure and TIM predicting exposure through multiple routes. Some of the major differences between the two models with relevance to hummingbird–insecticide risk assessment are reviewed below.

##### Initial Concentrations on Diet

For dietary exposure, total dietary dose can be estimated by multiplying food intake rates ($IR_{nectar}$, $IR_{invert}$, etc.) by pesticide concentrations in insects, nectar and pollen [52]. Equation (1) gives an example for a bird (e.g., a hummingbird) that consumes only nectar and invertebrates.


(1) $$D_{diet(t)}=C_{nectar(t)}*IR_{nectar}+C_{invert(t)}*IR_{\text{i}nvert}$$

T-REX predicts initial pesticide residues on classes of dietary items (short grass, tall grass, broadleaf forage, seeds, and fruits) using nomograms developed from empirical studies of a variety of pesticide–crop combinations [53,54]. Residues on invertebrates are similarly predicted using data from empirical studies [45]. Pesticide initial concentrations on dietary items are modeled as the product of the application rate and the nomogram value corresponding to each dietary class, summed across dietary classes. TIM predicts avian exposure resulting from realistic time-dependent pesticide use scenarios. Detailed information on TIM has been provided elsewhere [34,38], and we include a brief summary of important features here. TIM accounts for exposure through diet, drinking water, inhalation, and dermal contact following insecticide spray application to crops using a 1 h timestep. Nondietary routes of exposure are converted to dietary equivalents to estimate total dose. The distributions of initial residues on dietary items following exposure are assumed to follow lognormal distributions [54] normalized to 1 lb active ingredient/acre and multiplied by the application rates in similar fashion to T-REX nomogram values.

Unlike T-REX, TIM also simulates exposure adjacent to a treated field using an approach adapted from the AgDRIFT model [34]. Parameters that affect spray drift in TIM include distance from the treated field, the use of an in-field buffer, spray application type, and droplet size. Drift exposure is assumed to be limited to 303 m from the field edge and is expressed as a fraction of the corresponding values from the treated field. Birds are assumed to forage on/off fields according to a Markov transition matrix with stable distribution for time spent foraging on field determined by the parameter “frequency on field” (details on convergence equations are given in the TIM online documentation). Drift exposure only occurs when the bird is foraging off-field and within 303 m, depending on parameterization. TIM can be set to simulate only off-field foraging to examine the effects of spray drift exposure alone.

Although neither T-REX nor TIM currently have nectar exposure methods, USEPA has approaches available to estimate pesticide concentrations in nectar. Methods for estimating pesticide residues in nectar have been developed and applied at USEPA for application to insect pollinators. These include methods for foliar spray, seed, and soil treatments [55]. Each of these could be incorporated (with some modification) into exposure models for avian nectar consumption. Lacking ready-to-use models, USEPA has modeled residues on nectar from spray applications using predicted residues on tall grass as a proxy [45,56], which we combine here with the assumption that RTHU consumes 50% nectar and 50% invertebrates during the breeding season [50]. The assumption that tall-grass residues are representative of pesticide residues in nectar is an important uncertainty that could be addressed using available empirical residue data [57]. User-defined nomograms are possible in the T-REX implementation in MCnest. However, reparameterization of the lognormal distributions of dietary residues is not currently permitted in the joint TIM–MCnest model.

##### Consumption of Contaminated Food

Metabolic processes in living organisms have repeatedly been shown to be well characterized with allometric relationships based on body mass [58]. Field allometric relationships between body mass and energy expenditures are typically developed to compare taxon-specific metabolic rates in the form of a generalized linear regression model with energy use as the dependent variable and log body mass as the independent variable. The value of the slope parameter provides information on the scaling efficiency of energy budget components within taxa. Birds are one of the most successful terrestrial taxa, covering more of the earth’s surface than any other vertebrate group, due to a host of evolutionary modifications relating to flight. These adaptations include rapid wing movement essential for flapping flight and the ability to generate mechanical power output for sustained periods. The amount of metabolic power necessary for flight is dependent on body mass, with smaller birds (e.g., hummingbirds) requiring a lesser multiple of their basal metabolic rate compared to larger birds (e.g., vultures). Of all the vertebrates, hummingbirds have the highest mass-specific metabolic rate [59]. Smaller birds can use these relative efficiencies to enable greater maneuverability and acceleration. These evolutionary pressures are particularly relevant for migratory birds and especially apparent in oxidative metabolism adaptations to meet energetic demands. Such relationships are important to understand when considering both exposure and effects for comparing birds to other taxa, but also for comparing different classes and life histories with Aves.

For many pathways, chemical exposures are correlated with metabolic activity patterns. Since flight imposes evolutionary pressures via high metabolic demands and lower body mass, birds generally have higher mass-adjusted daily energy needs when compared to other vertebrates. Birds meet these metabolic demands through food consumption. In agroecosystems, food resources often contain pesticide residues, whether they are nectar, treated seed, insects, or other resources. Within Aves, hummingbirds are the most extreme examples of high mass-adjusted daily energy needs.

Estimation of food consumption rates should begin by assuming consumption sufficient to meet daily energy expenditures. Energetic requirements vary throughout the avian life cycle, with high demand occurring during early growth and development, migration, reproduction, and molt [60]. However, seasonal variability in consumption can be challenging to quantitatively estimate. Adult metabolic rates account for all assumed costs of existence plus additional functions such as thermoregulation, locomotion, and feeding, while juvenile rates account for persistence alone, but differentiations between more advanced states are not captured beyond that. Avian basal metabolic rates (BMR) scale allometrically with body weight (see Equation (5) below), with b=0.68 [61,62]. Therefore, smaller species have higher mass-specific metabolic demands. Thus, the energetic demands of costly periods of the life cycle are likely to be especially acute for hummingbirds and it is noteworthy that migration is relatively rare in the family and that clutch sizes are small. These periods of energetic demand must be offset by increased consumption, potentially greatly increasing hummingbird exposure to contaminants relative to other birds feeding on similar resources.

In T-REX, avian daily food consumption rates (F) are assumed to follow an allometric relationship with body weight (BW, Equation (2)) while controlling for consumed food water content (W). Total dose on application day can then be estimated as the weighted (by proportion of diet) sum across all dietary classes of the residues on dietary items and the amount consumed subject to the constraint that total consumption equals the food intake rate (F).


(2) $$\text{F}=\left(0.648*{\text{BW}}^{0.651}\right)/(1-\text{W})$$

In TIM, food intake is governed by a reported field metabolic rate (FMR, kcal/bird/day), the equation for which differs for passerines (higher) versus non-passerines and for juveniles (lowest). Birds are assumed to meet the FMR demand through consumption of dietary classes (arthropods, seeds, fruit, grass, and broadleaf forage) with specific known concentrations of metabolizable energy (ME, kgal/g food). Total daily food intake (g food/day) is then calculated as the quotient of FMR and ME, to which a stochastic error is applied to vary intake by ±10%. Users can modify the daily intake rate through application of a gorging factor to account for periods of high energetic demand.

##### Pesticide Degradation

Pesticide concentrations on dietary items at different times are calculated in both T-REX and TIM using versions of Equations (3) and (4), conditional on initial residue values following application and assuming a first-order exponential decay function. Foliar dissipation half-life values $\left(t_{\frac{1}{2}}\right)$ can be obtained from the literature or from registrant submitted studies. A default value of 35 days is generally used when no pesticide-specific data are available [34,45,63].


(3) $$C_{diet(t)}=C_{diet(t=0)}*e^{-kt}$$

(4) $$k=ln(0.5)/t_{1/2}$$

##### Other Exposure Routes

As noted above, T-REX predicts exposure only through diet, whereas TIM considers exposure through other sources, including dermal contact, drinking water, and inhalation. These methods are all readily applicable to hummingbirds as well as other taxa, with dose estimated in dietary equivalents and added to dietary dose. Online documentation for the TIM model offers detailed explanations of the equations and assumptions underlying these methods.

##### Other Application Methods

The methods reviewed above for estimating initial pesticide residues on dietary items are specific to foliar spray applications. However, pesticides may also be applied through other means including trunk injections, seed, and soil treatments. For insecticides with low water solubility, these application methods may pose relatively low risk to hummingbirds, compared to granivorous birds and/or those that pick grit. However, water-soluble insecticides have the potential to be systematically expressed and/or absorbed by plants and transported to tissues that are consumed by birds (e.g., fruit, nectar). Many neonicotinoids, including imidacloprid, have relatively high water solubility. However, comparative analysis of nectar and pollen residues following foliar spray versus soil treatments showed much lower residues associated with the latter [57], indicating that risk may be low. Further development and inclusion in the avian toolbox of methods for predicting nectar concentrations for water soluble chemicals is an important area for model development to support higher-tier risk assessment for hummingbirds.

##### Summary Assessment of Existing USEPA Exposure Methods for Hummingbirds

In summary, TIM offers several advantages over T-REX for hummingbird exposure modeling. It includes additional exposure pathways other than diet, the ability to model exposure in adjacent habitats, which likely have more preferred nectar sources than crops, and it takes an explicitly bioenergetic approach to modeling. However, it also has some important limitations. Like T-REX, nectar is not a specific dietary class available in TIM. Nor can users modify the lognormal distributional parameters governing initial residues on dietary items to co-opt an unused class for nectar residues. Similarly, users cannot modify drift distances or bioenergetic requirements beyond the gorging factor. Finally, for hummingbirds, preening may also be a route of exposure [9], which cannot currently be modeled in TIM. Neither TIM nor T-REX, as currently implemented in the MCnest model, offer methods for soil or seed treatments.

#### 3.3.2. Effects

Given similar exposures, the effects of pesticides on different bird species may vary with many factors, including genetics or physiology of the potentially affected cellular or organ-level processes. These endogenous processes are captured in adverse-outcome pathways (AOPs, [64]), though interspecies variability in AOPs in birds has been little explored. Quantitative AOPs [65] can function as mechanistic dose–response models [66] that could be used in MCnest in place of the threshold effects the model currently uses [20]. Effects may also depend on the ability of birds to eliminate the pesticide through egestion and/or metabolism, and birds with higher clearance rates may therefore be at lower risk. Clearance may depend on metabolic rates, as noted above, making birds with high metabolic rates, like hummingbirds, potentially less vulnerable. Individual fitness in response to exposure also depends on an interaction between the type of adverse effect (survival or reproduction) caused by the chemical and the endogenous life cycle of the bird [20]. The life cycles of US hummingbirds strongly resemble those of other small birds, generally being on the “fast” end of the life history spectrum (short life span, early maturation). However, hummingbirds, and other nectarivores, are noteworthy in this group for having relatively low mass-specific reproductive success [67].

##### Adverse-Outcome Pathways (AOP) and Dose–Response

MCnest expands the RQ concept by comparing dynamic modeled exposure to surrogate toxicity endpoints [49,68] on a daily basis while the stochastic breeding model is running. Surrogate endpoints are designed to be phase-specific (e.g., egg development, egg laying, incubation, nestling care) and are chosen carefully from the suite of measured endpoints from the toxicity tests. (e.g., eggshell thinning, number of viable eggs set per hen, number of hatchlings produced per viable egg, etc.). A nest attempt is assumed to fail if the appropriate exposure measure (derived from T-REX or TIM, depending on which exposure model has been run) exceeds the surrogate endpoint. These generalized RQs used as surrogate endpoints were chosen because they are commensurate with the data from standardized toxicity tests. Generalizing these threshold response functions using AOP or dose–response modeling might increase the utility of MCnest for higher-tier risk assessment for hummingbirds.

Insecticides are a class of chemicals for which AOPs have been, and could be further, developed (e.g., [69]). In general, pesticides are developed with specific, intended, molecular initiating events (MIE), often, but not exclusively, targeting the neuromuscular system [70]. These biochemical pathways are often conserved in vertebrates, though the downstream key events resulting from the MIEs may, and likely do, differ from invertebrates. Etterson and Ankley [20] investigated an AOP for aryl hydrocarbon receptor activation in birds leading to embryonic mortality and reduced reproductive success [66]. Their work showed that the MCnest environment is compatible with an AOP framework, though they stopped short of providing a generalized method that could be easily implemented. Thus, further work to develop compatibility between MCnest and AOP, when the latter are available, could offer advantages over the phase-specific threshold exceedance model currently implemented in MCnest. Similar comments could be made about traditional dose–response models when available, which currently cannot be implemented in MCnest, outside of the step-function threshold models.

##### Metabolism and Toxicity

As noted above, there is a very general and robust relationship between body weight and metabolic rate among organisms [59] and within birds [71].


(5) $$BMR=a*bwt^{b}$$

In Equation (5), a=0.85 and b=0.68 for hummingbirds [72]

Mineau et al. [72] showed that risk of acute pesticide poisoning in birds is also allometrically related to body weight, with smaller birds at greater risk. For a variety of pesticides, they showed that toxicity, as measured by the median lethal dose (LD50), was related to body weight according to Equation (6).


(6) $$LD50=c*bwt^{d}$$

Together, Equations (5) and (6) define a third allometric relationship between toxicity and metabolic rate (Equation (7))

(7) $$LD50=k*BMR^{\left(\frac{d}{b}\right)}$$

**Figure 1.**
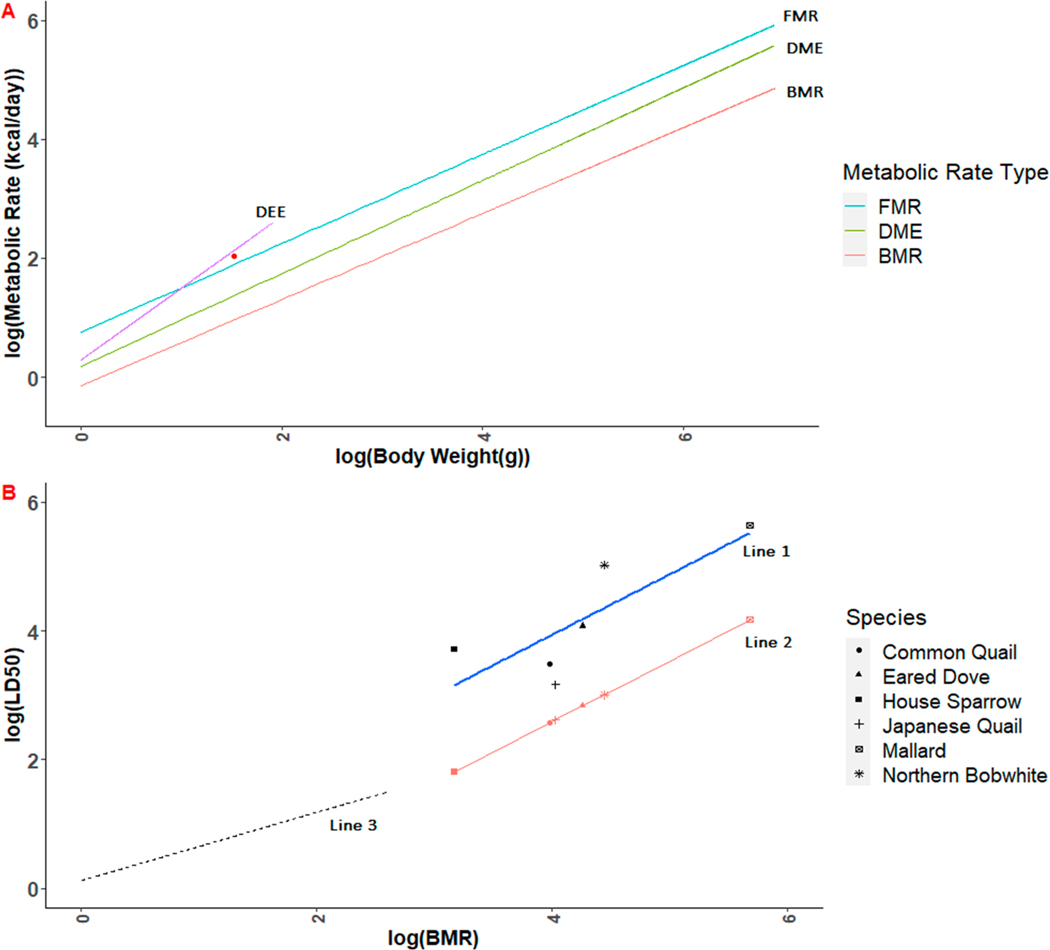
(**A**) shows the log–log relationship between metabolic rates used in the terrestrial investigation model (TIM) for basal metabolic rate (BMR), daily metabolized energy (DME), and field metabolic rate (FMR) as well as daily energy expenditure (DEE) for simulated body weight ranges for the ruby-throated hummingbird (*Archilochus colubris*). Powers and Nagy [15] detail a mean metabolic rate for the Anna’s hummingbird (*Calypte anna*), plotted here against the DEE. DEE is calculated using the relationship log(DEE) = 1.72 + 1.21 ∗ log(mass), from [72] BMR, DME, and FMR are calculated using standard allometric relationships between body weight and energetic requirements, detailed in the TIM Ver. 3.0 guidance, Appendix F [34]. (**B**) shows the log–log relationship of reported and estimated LD50s for imidacloprid and BMRs for several species and ruby-throated hummingbird. Line 1 (blue) corresponds to the fitted linear regression between reported LD50s for 6 species and their estimated BMR, using Equations (5) and (7). Line 2 (red) is the estimated LD50s using the fitted regression coefficients in Equation (6). Line 3 (purple) represents the estimated LD50 for hummingbirds across the range of body weights for US hummingbird species using slope and intercept values from [72] with regression coefficients derived from the fitted regression from Equation (7).

In Equation (7) $k=c*a^{-\left(\frac{d}{b}\right)}$. Equation (7) shows that, in general, tolerance should increase exponentially with metabolic rate, with allometric slope d/0.68. Empirical results for Equation (6) fit to 37 pesticides provide values for d ranging from 0.6284 (diazinon) to 1.5370 (nicotine sulfate), with all but one value (diazinon) > 0.68 [72]. Therefore, for most pesticides, according to Equation (7), the LD50 will be an accelerating function of BMR. Noting that k in Equation (7) is necessarily positive, birds with a positive residual on the allometric equation for BMR (Equation (5)), such as hummingbirds, are predicted to be less sensitive (higher LD50 predicted by Equation (6)) than those that follow the expected relationship or have a negative residual. In other words, in the combined equation, hummingbirds would have a larger apparent body weight (Equation (5)) due to their exceptional metabolic rate, resulting in a higher predicted LD50 according to Equation (7). Thus, if combining the two allometric equations is valid, then hummingbirds are likely to be less sensitive than would a bird with similar body weight, but lower metabolic rate (Figure 1A).

**Figure 2.**
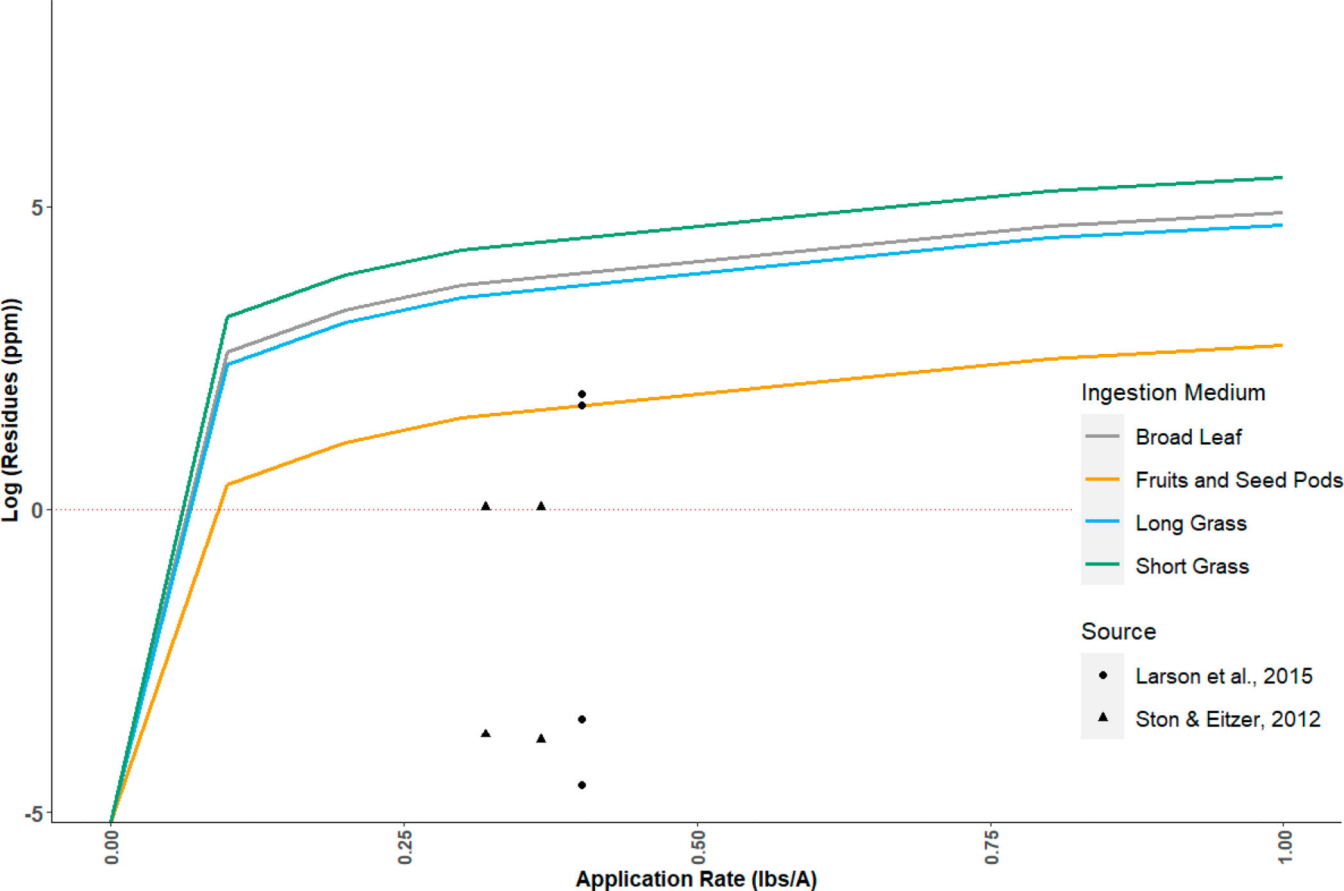
Natural log of estimated imidacloprid residues using the terrestrial residue exposure (T-REX) model guidance for dissipation of a chemical applied to foliar surfaces with ingestion mediums used in MCnest. Nectar residues for imidacloprid spray reported in [73,74] are plotted for comparison against the modeled residues. The model results are biased high as expected since T-REX is intended for screening assessment.

The above considerations highlight several important considerations for the bioenergetics of avian consumption and clearance. They suggest that both T-REX and TIM are conservative with respect to the adverse effects of imidacloprid on hummingbirds, though for different reasons. T-REX, as intended, likely overestimates exposure (Figure 2), consistent with its use as a screening level model. TIM likely underestimates the LD50 for hummingbirds (Figure 1B). In TIM, users can modify intake rates only by specifying a species as being either passerine or non-passerine, resulting in different estimates of the field metabolic rate for a given body weight, or by modifying the gorging factor, resulting in a linear scaling of consumption. No user control is provided over the metabolizable energy in food resources. More control is available to the user to modify clearance rates, which are modeled using a first order decay function with parameter “hourly fraction of pesticide retained.” Thus, the extreme metabolism of hummingbirds and associated increased consumption and clearance would be difficult to simulate in the current MCnest model.

##### Life Cycle

Ecologists have long held out hope that life history theory could provide insight into the susceptibility of different species to anthropogenic disturbance [75]. Birds, like many other taxa, are well characterized along a fast–slow gradient, with “fast” species showing low survival rates, early maturation, and, often, high fecundity [76]. In contrast, “slow” species have long life spans and late maturation. Hummingbirds exhibit a typical fast life history and as such are likely most sensitive to stressors that negatively influence early life mortality [20]. Such organisms are likely able to recover quickly, following disturbance, assuming the stressor is removed. The ability to incorporate life cycles and life history theory into model construction is a strength of MCnest [29,51].

#### 3.3.3. Population and Behavioral Factors

Assessing avian population and spatial factors influencing pesticide exposure within a spatially, temporally, and behaviorally explicit context is challenging. Published methods primarily focus on acute and/or chronic exposure risk in a spatial context, as integration of population-level factors is complex and species-dependent [77–79]. Spatial factors may be of particular importance for hummingbirds: while foraging patterns and preferences can vary depending on species, hummingbird responses to landscape structure often reflect habitat quality and quantity, behavioral traits, and prior experience. Baum et al. [79] found that hummingbirds may apply unique foraging rules depending on landscape patch types. For example, random, uniform, and clumped landscape structures resulted in differing directional strategies, suggesting complex interactions between hummingbirds and habitats, as well as implications for exposure scenarios. Little evidence supports the common use of agricultural fields as a primary foraging resource for hummingbirds in the US [80], yet exposure via use of edge habitats or landscapes within drift zones is likely. Bishop et al. [7] documented residues of three neonicotinoids in cloacal fluid of rufous (*Selasphorus rufus*) and Anna’s hummingbirds within 0.5–1 km of sprayed blueberry fields in Canada. Additionally, neonicotinoid pesticides were detected in cloacal fluid of hummingbirds captured up to 22.8 km from sprayed blueberry fields across temporal patterns that suggest chronic exposure [81].

Temporal exposure factors affecting fecundity depend on timing of relevant agricultural exposures and of pre-breeding and breeding behavior. Northward migration of RTHU immediately prior to the breeding season likely follows trends in nectar flows starting in early spring [82]. Evidence suggests that RTHU may utilize the presence of sap wells created by the yellow-bellied sapsucker (*Sphyrapicus varius*) and in some cases feed almost exclusively on tree sap, indicating that nectar and pollen are consumed less prior to and during breeding [83]. This has implications for the timing of exposures, as individuals foraging primarily on sap during breeding may avoid direct consumption of contaminated nectar. However, this pattern may not be universal. Hummingbird cloacal fluid collected in early spring suggests exposure to pesticides during migration [81]. Thus, for migratory hummingbirds, exposure in relation to the timing and location of wintering sites, migratory stopover, and breeding locations is likely to be both complicated and highly variable among populations.

Preferences between nectar and sap are still relatively uncharacterized. Southwick and Southwick [83] suggest that foraging efficiency is increased greatly via sap, and nectar did not appear to be a primary food source even when available. Much less is known about insect foraging, and the role of insect dietary preference as it relates to timing of breeding (but see Moran et al. [84]). However, hummingbirds utilizing sap resources have been seen to feed on insects directly from trees [82], suggesting that reliance on or preference for sap and reduction of nectar intake may coincide with increased insect consumption, further complicating the task of estimating dietary exposure.

Documented behavioral traits among hummingbirds may also influence the magnitude and breadth of exposure for individuals. Color preferences and plant morphology have been linked to specific foraging behavior in hummingbird species [85]. In addition, hummingbirds are highly territorial, displaying resource defense strategies that can be dictated by visibility of food resources, habitat type and quality, and seasonality [86]. Altogether, evidence suggests that these species may undergo complex exposure scenarios relevant to fecundity that are challenging to model in any framework.

MCnest’s capacity to include spatial, temporal and behavioral components is limited. Users can specify parameters in TIM denoting fraction of edge habitat receiving spray drift, the width of in-field buffers, and the frequency with which a bird forages on treated fields, but the model does not specify explicit landscapes or locations or specific foraging preferences beyond intake. MCnest predictions have been previously used within the HexSim modeling environment to simulate a spatially explicit population model for the California gnatcatcher, *Polioptila californica* [51]. The HexSim environment allows for inclusion of multiple spatial components, including habitat, insecticide use, and patch landscapes. Future efforts to include relevant spatial aspects could combine MCnest outputs in the HexSim modeling environment with appropriate patch landscapes and habitats suitable for hummingbird-specific exposure scenarios.

### 3.4. Conceptual Model (Phase 4)

**Figure 3.**
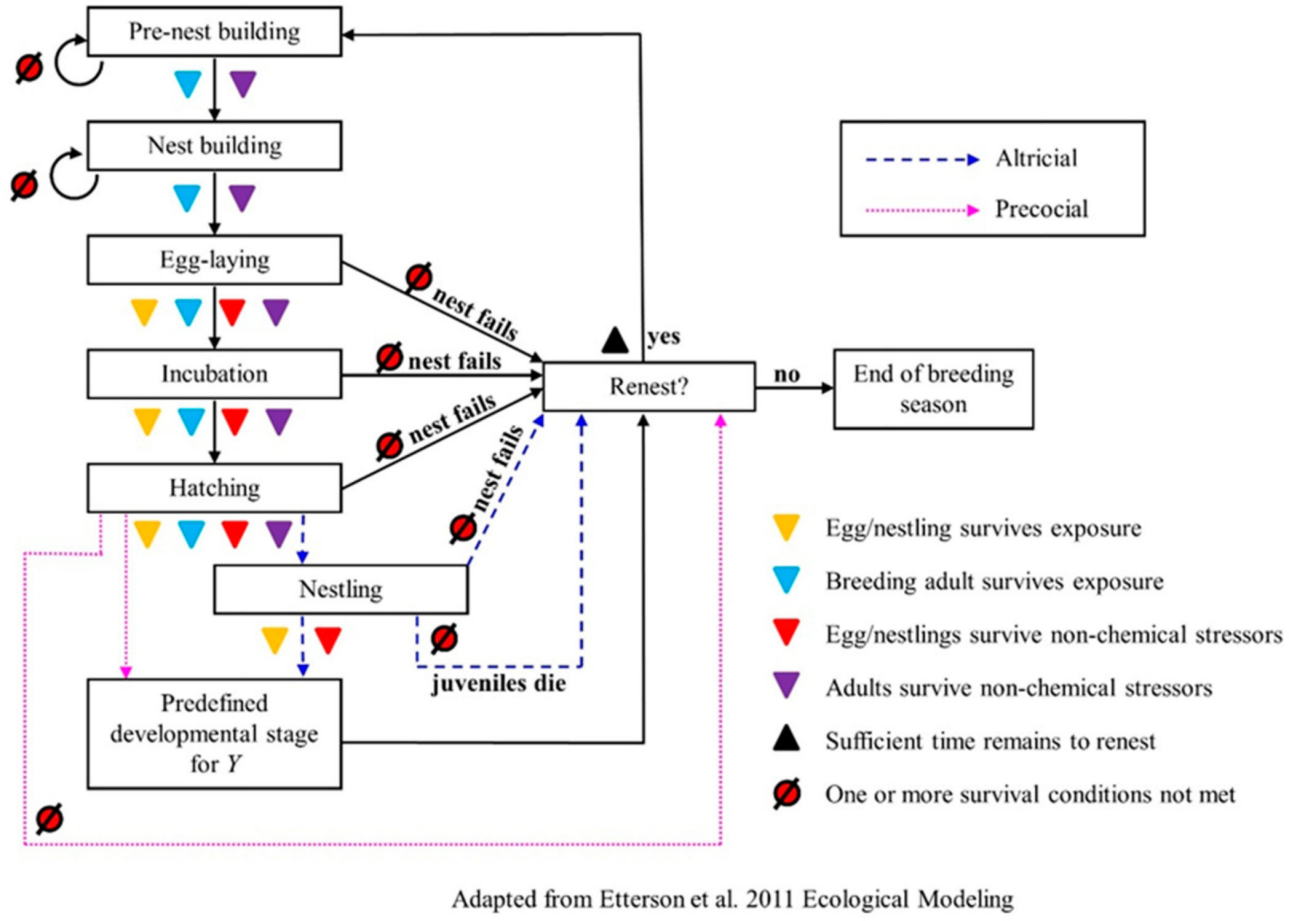
Conceptual model showing how chemical and other stressors are mapped onto an iterative model of avian seasonal reproduction. Y refers to the desired output from the breeding model, which is user-defined and could be number of successful broods, number of offspring produced, the probability of at least one successful brood, etc. Adapted from [29].

The fourth phase of the Pop GUIDE model design is to create a conceptual model showing how the algorithms and objectives described above could be knitted together to conduct an assessment. In this case, we evaluate MCnest as a potential unifying model. We present the MCnest conceptual model [29] modified to indicate where the exposure and effects pieces would influence the dynamical process (Figure 3).

MCnest represents the avian breeding cycle using an iterative algorithm describing typical species-specific progression through a series of developmental stages (ovum development, egg formation, egg laying, incubation, and nestling care) (Figure 3, [29]). The algorithm is coded as a Markov Chain transition matrix [87] and incorporates species-specific propensities to renest after either nest failure or nest success to predict the seasonal productivity of breeding females under alternative exposure conditions. This design allows MCnest to be readily and quickly reparameterized for many different avian species. Virtually all socially monogamous temperate birds can be modeled using this algorithm, though some mating systems, such as serial polyandry, could not be easily accommodated.

### 3.5. Model Implementation and Evaluation (Phase 5)

The fifth Pop-GUIDE phase includes model implementation as a computational tool and evaluation of its performance. This step includes model parameterization, execution, and calibration as needed. The evaluation component then assesses output behavior and compares predictions to available data. Our review has identified multiple limitations in the ability of the MCnest/T-REX/TIM modeling system to fully capture all relevant details for refined pesticide risk assessment for hummingbirds. Therefore, a limited illustration using the model in its current form is provided.

For MCnest predictions, a single application was modeled and compared across pesticides and species. Screening level MCnest predictions were generated using T-REX as the exposure algorithm and assuming birds forage only on treated fields. More refined assessments were then conducted using TIM as the exposure algorithm by modifying MCnest species profiles assuming RTHU only use adjacent edge habitats (maximum frequency on field = 0.05) to evaluate the potential for unintended exposure to affect birds. For consistency, this assumption was applied to all species, though many species in the MCnest library are known to use agricultural fields during the breeding season [88]. Dietary residues resulting from foliar spray applications were estimated and modeled as described above for all pesticides. All comparisons use the expected fledglings/female/year as the model prediction expressed as percentage reductions under exposure conditions compared to unexposed.

#### 3.5.1. MCnest Illustration

Application rates (lb a.i./A) for imidacloprid, indoxacarb, λ-cyhalothrin, and permethrin applied to soybeans were taken from the PPLS (Table 3). Application data for chlorpyrifos and methomyl were taken from recent USEPA Endangered Species Biological Evaluations [39,40]. Application rates represent currently registered products containing these active ingredients.

For MCnest simulations, initial residues on invertebrates from spray applications were modeled following the analysis described in Appendix B of the T-REX manual [33]. Accordingly, an initial residue unit dose (RUD = mg a.i./kg arthropod/lb a.i. sprayed/acre) of 94 RUD was multiplied by the application rate to determine initial arthropod concentrations. These concentrations were assumed to decline following the same exponential decay described in Equations (3) and (4), according to the foliar dissipation half-life, calculated following recommendations in [33], Appendix A. For simulations using TIM, the initial distribution of pesticide on arthropods was drawn from a lognormal distribution with mean = 65 and SD = 48 RUD. As with T-REX, dissipation of residues on arthropods were modeled according to a first order exponential decay, determined by the chemical-specific foliar dissipation half-life.

**Figure 4.**
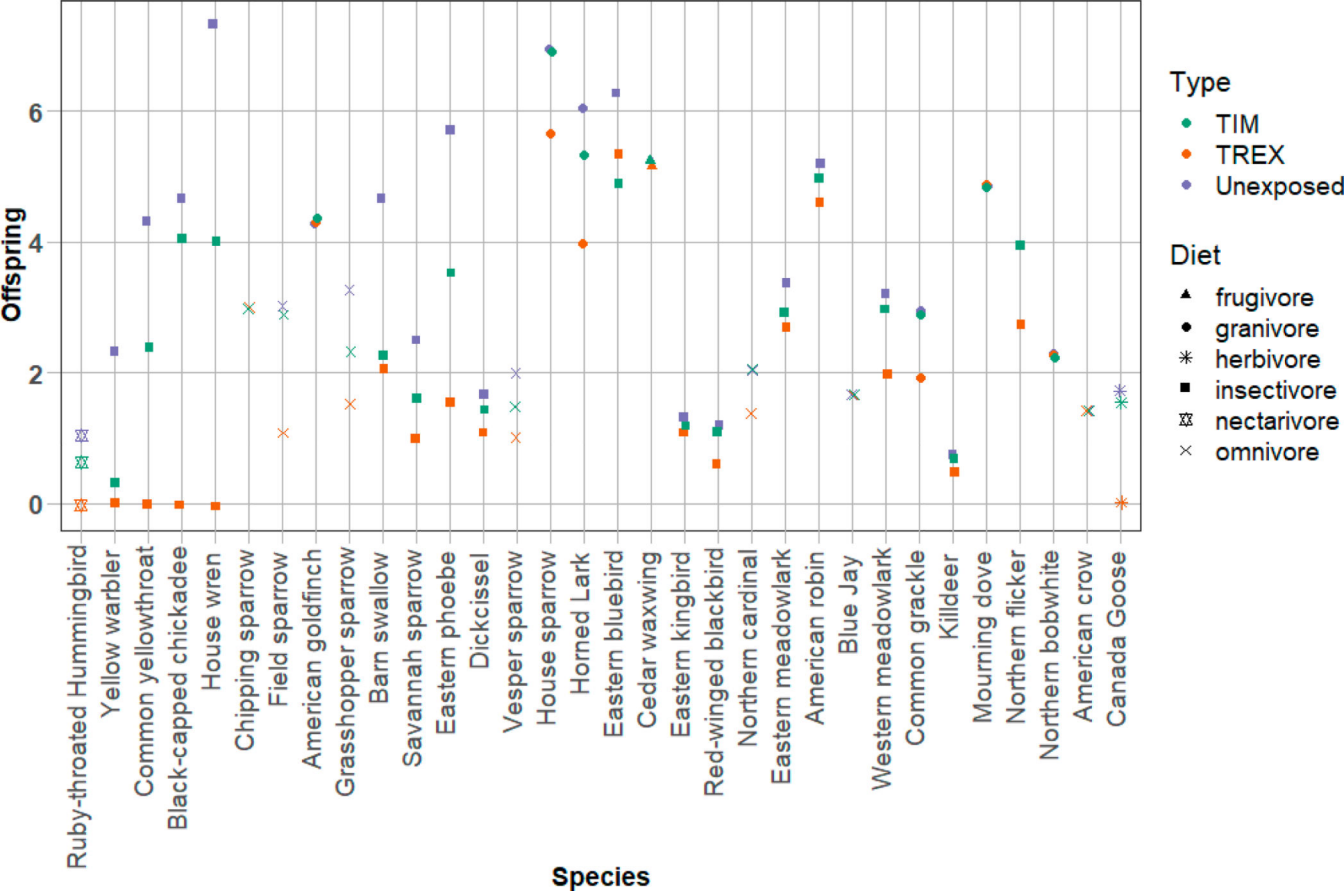
Estimated fecundity (offspring/breeding female/year) for 32 avian species simulated in MCnest using TIM, T-REX, and an unexposed scenario. Diet (frugivore, granivore, herbivore, insectivore, or omnivore) is determined using the MCnest feeding classes based on proportion of dietary intake; frugivore and nectarivore are added as an additional category here for species primarily foraging on fruits or nectar. The species are ordered from lowest (ruby-throated hummingbird) to highest (Canada goose) mass in grams.

MCnest predictions suggest that hummingbirds in and near agricultural fields treated with imidacloprid are likely to experience some adverse effects of pesticide exposure. Using TREX (which assumes that RTHU forage only on treated fields) as a screening level exposure algorithm, MCnest predicted 100% reduction in RTHU reproductive success, as measured by the number of fledglings/female/year (Figure 4). Five other species also had predicted reproductive success reductions of 100% and the remainder ranged down to no effect (0% reduction, Figure 4). Simulations using TREX assumed only reproductive effects and no adult mortality.

In contrast, MCnest using TIM, in which birds were assumed to use only edge habitats adjacent to treated fields and therefore receive exposure only through spray drift, predicted reduced or no impacts (Figure 4). This was true despite the added possibility of adult mortality modeled by TIM using the empirical LD50 and slope. No species experienced complete reproductive failure and the maximum observed effect was a 21% reduction (grasshopper sparrow, *Ammodramus savannarum*). RTHU was predicted to experience an 18% loss of reproductive success, the third highest proportional effect of all modeled species (Figure 4). Predicted RTHU adult mortality due to pesticide exposure was about 4%, accounting for roughly a quarter of the observed reproductive failure.

Table 4 shows the predicted effects on RTHU of exposure to a single application of each of six pesticides following labeled guidelines for use on soybeans. Except for chlorpyrifos (for which registered uses of chlorpyrifos on food crops are subject to cancellation under the Federal Insecticide, Fungicide, and Rodenticide Act), imidacloprid is predicted to have the highest effect on RTHU reproductive success following a single application, followed closely by methomyl. For three of the six modeled pesticides, a single application seemed to pose negligible (~1% loss) risk to reproductive success of RTHU if their foraging activity is restricted to habitats adjacent to fields (i.e., birds foraging on fields less than 5% of the time). However, this conclusion should be interpreted with caution. The extent to which RTHU forage on agricultural fields remains uncertain. These simulations only explore a single application occurring on a fixed date during the breeding season. However, birds would likely be exposed to multiple applications and dates of applications would vary relative to the timing of the season.

The T-REX simulations presented here may also underestimate RTHU exposure because hummingbirds have higher metabolic demands than predicted based on the standard avian allometric equation (Equation (5)). While we could not find data specific to RTHU, a bioenergetic study of the closely related Anna’s hummingbird (*Calypte anna*) estimated that they require 9.9 g of nectar to meet daily energy expenditure requirements [15]. Taking nectar sugar content to be approximately 40% [89] and water content to be the complement of sugar content (i.e., 60%), the USEPA [33] food intake equation (Equation (7)) predicts that a 4.6 g Anna’s hummingbird feeding exclusively on nectar would consume 4.4 g, 56% less than that predicted by Powers and Nagy [15]. Thus, the T-REX model, which is intended to be conservative, might underestimate nectar consumption. However, using tall grass as a proxy for nectar, with a default assumption of 80% water content, results in only a slight underestimate (12%) of predicted nectar consumption. In contrast, TIM uses a bioenergetic approach to estimate dose [34] that appears to correspond reasonably closely to available daily energy expenditure (DEE) requirements for Anna’s hummingbird (Figure 1A). More generally, nectar sugar content is highly variable, with some studies (e.g., [90,91]) finding sugar content between 16–28% in hummingbird frequented plants in the tropics. Thus, a generalized methodology for risk assessment for avian pollinators would be strengthened by a nectar-specific nomogram and user control over the food consumption equation to accommodate higher-than-expected metabolic rates and associated food consumption, as previously noted. As noted above, another model improvement would be to allow user input of empirical residue data when available [58].

#### 3.5.2. Sensitivity Analysis

To help identify potential uncertainties and knowledge gaps, a local sensitivity analysis was conducted on the RTHU model predictions by perturbing model parameters ± 5% and comparing the resulting magnitude of change in predicted fledglings/female/year. Sensitivity analyses were conducted only for MCnest simulations with TIM and the sensitivity metric used was the discrete approximation to elasticity calculated by substituting $\text{Δ}x=x-x^{\prime }$ and $\text{Δ}y=y-y^{\prime }$ for dx and dy in Equation (8). In Equation (8), y represents fledglings/female/year, x represents a parameter chosen for perturbation, and the prime symbol indicates the perturbed value for each. Both forward and backward perturbations of 5% were calculated and the average was taken as the sensitivity measure.


(8) $$elasticity(x)=\frac{x}{y}\frac{dy}{dx}≅\frac{x}{y}\frac{\text{Δ}y}{\text{Δ}x}$$

Only one model parameter showed an elasticity magnitude greater than 1. When this is the case the magnitude of the slope of the response with respect to the given model parameter is larger than the ratio of response to parameter $\left(|elasticity(x)|>1\to \left|\frac{\text{Δ}y}{\text{Δ}x}\right|>\left|\frac{y}{x}\right|\right)$ Therefore, any such parameter is disproportionately influential to the model response, generating an ever-widening response divergence with perturbation. In contrast, when elasticity<1, parameter perturbations result in a disproportionately smaller change in the response variable and tend to dampen the ratio of response to parameter with greater perturbation. The full set of elasticities is reported in the Supplementary Material (Table S2).

The single parameter showing a disproportionately large influence (∣elasticity∣>1) was the fraction of pesticide available from one hour to the next (elasticity=−12.3), an order of magnitude larger than the next most important parameter, the Mineau [72] scaling factor (elasticity=−0.93). Other highly influential parameters (∣elasticity∣>0.5) included two life history parameters (the lengths of the nestling and incubation periods) and two toxicological parameters (the slope of the avian oral LD50 and the pesticide application rate). Three of the four influential toxicological parameters describe the toxicity and toxicokinetics of imidacloprid, though the Mineau scaling factor also introduces life history via body weight. Three of these influential toxicity parameters, the fraction of pesticide retained from one hour to the next, the Mineau scaling factor, and the slope of the LD50 are also parameters about which there is substantial uncertainty concerning their empirical value, reviewed below.

The TIM parameter “fraction of pesticide retained from one hour to the next” is typically calculated from the Residue Chemistry Test (OPPTS 860.1480, [92]) using the domestic chicken (*Gallus gallus*) as a study organism. For imidacloprid, the value obtained from that study is 0.974, similar to the same values reported for the five previously published parameter sets [37], which ranged from 0.912 to 0.998. However, English et al. [18] reported an elimination half-life for RTHU for imidacloprid as 2.1 h, resulting in an estimate of the hourly fraction retained of 0.719, which is substantially lower and suggests much faster elimination kinetics for RTHU and imidacloprid compared to the other five pesticides modeled herein. Because the elasticity for the fraction retained is negative, a lower value would result in higher predicted fecundity. Biologically, this may be plausible given the extreme metabolism of RTHU. It is also supported by the short imidacloprid elimination half-life reported in rats (3 h, [93]). However, differences in experimental methodology between the RTHU study, which was not conducted following the USEPA [92] guidelines, may also have impacted the estimate. Thus, there is substantial uncertainty concerning the best value for predicting effects on RTHU versus comparing the relative potential risk of different pesticides. If the lower value for RTHU is due to their very high metabolism, then the elimination rates for the other pesticides are also likely to overestimate the fraction retained of those pesticides for RTHU. Given the high influence of this parameter, this is an important area for future research.

The Mineau scaling factor is typically taken from the primary reference [72] when risk assessment is for a chemical included in that study. Otherwise, the default value of 1.15 is used [34]. We replicated the Mineau et al. [72] methodology using six available estimates of the LD50 (Supplementary Material Table S3). Regressing log(LD50) on log(female body weight) resulted in a non-significant slope estimate of 0.641 (p=0.053). However, this p-value is for a test of whether the slope differs from zero. A more appropriate test in this case might be to test whether the slope differs from 1.15, which is the value that would otherwise be used. Accordingly, a one-sided t-test with 4 degrees of freedom gives p=0.048, suggesting that the default scaling parameter of 1.15 is too high for imidacloprid. Because fecundity has a negative elasticity for the scaling factor, a smaller Mineau value would result in smaller reduction in fecundity, holding all other parameters constant. Like the elimination rate described above, the best allometric scaling factor for imidacloprid and other neonicotinoids is an important area for future research.

The slope of the LD50 may also have substantial uncertainty. For example, Hill et al. [94] found substantial variation in slope of the LD50 for diazinon among groups of northern bobwhite (range 4.0–9.0), though they also found little difference in the expected value of the LD50. Thus, use of the slope of the LD50 to characterize the distribution of toxic sensitivity carries more uncertainty than does the median lethal dose as a measure of central tendency. While this parameter is uncertain, we have no supporting information to suggest it is biased either high or low.

## 4. Future Directions and Conclusions

The methods reviewed and implemented herein are a modest beginning toward the goal of a higher-tier modeling system for understanding the risk of pesticide exposure to populations of hummingbirds and other avian pollinators, such as Hawaiian honeycreepers (Fringillidae; Carduelinae), sunbirds (Nectariniidae) and Australian honeyeaters (Meliphagidae). Recent ecotoxicological research has highlighted the need for a comprehensive suite of models and methods for estimating pesticide effects on populations that account for risk assessment objectives, context, and available data [28]. These methods must be flexible enough to be implemented within a tiered system for ecological risk assessment [95] that optimizes limited resources. Although the MCnest system is currently oriented towards FIFRA-based pesticide registration decisions, Etterson and colleagues [51] showed how it could be implemented at different tiers, depending on objectives and available data. Future development of the MCnest model should focus on expanding model flexibility and giving greater user control over the structure and parameterization of its modules.

To our first question, whether hummingbird exposure and effects can be modeled using existing risk assessment tools in the USEPA avian risk assessment toolbox, our answer is both yes and no. We were able to adapt existing models to produce a preliminary assessment for foliar spray applications of pesticides that gives valuable insight into relative risk among pesticides and in comparing hummingbirds to other birds. However, our review highlights many important limitations and areas for future research and model development. These include research on the importance of metabolic considerations in determining exposure and effects of pesticides, especially for birds, such as hummingbirds, that do not closely conform to the usual allometry of body size and metabolic rates. Models like MCnest must also be generalized to include a wider range of pesticide application methods such as seed treatments and soil applications, and our review identified methods and algorithms that could be used for this purpose (Supplementary Material). Future research should also focus on the extent to which hummingbirds may be exposed to pesticides through other pathways, including dermal contact, inhalation, and drinking water. Because it is impossible to anticipate all possible contexts and relevant data, MCnest should be generalized to greater modularity and to give greater flexibility for incorporating user-defined submodels. Our review also identifies the need for greater flexibility to incorporate spatial considerations in avian risk assessment tools. These and other areas for future research highlighted throughout this paper address our second question concerning what enhancements to the current toolbox would make hummingbird risk assessment more realistic and more accurate.

Notwithstanding the limitations described above, this review also addresses our third question concerning what risk predictions do current tools make about hummingbird risk and how this compares to other species and between pesticide classes. Our work supports the conclusion that hummingbirds are among the species using agricultural ecosystems that may receive higher exposure to pesticides. Conservative screening level assessments with T-REX suggested the potential for effects and more refined estimates using the TIM model suggested that RTHU would be among the species potentially affected by spray applications of imidacloprid. Comparing among chemicals, imidacloprid potentially has greater impacts on fecundity than four of the other five pesticides modeled. However, we emphasize that this is a preliminary indication that should be further investigated. This work showed several key areas of uncertainty when using available pesticide exposure and toxicity information, simulation models and species life history information to estimated effects on hummingbirds. Although our implementation focused on selected neonicotinoids, these results are highly relevant for any pesticide appearing in nectar, particularly for seed treatments or GM-related plant incorporated protectants that are expressed in nectar. Further investigation of the most influential assumptions and parameters could improve our ability to estimate potential effects of pesticides on the reproductive success of hummingbirds, including relative comparisons among pesticides.

## Supplementary Material

Supplement1

## Figures and Tables

**Table 1.** MCnest life history parameters for ruby-throated Hummingbird.

Parameter	Value

Date of first egg in first nest	24 May
Date of first egg in last nest	22 July
Length of rapid follicle growth (d)	2
Egg laying interval (d)	2
Length of incubation period (d)	17
Length of nestling period (d)	18
Breeding initiation probability (d^−1^)	0.25
Nest failure rate during incubation (d^−1^)	0.03
Nest failure rate after hatch (d^−1^)	0.03
Adult survival rate (d^−1^)	0.9976
Proportion of diet consisting of nectar	0.5
Proportion of diet consisting of invertebrates	0.5

**Table 2.** Representative avian toxicity test results for birds exposed to imidacloprid.

Toxicity Endpoint	Value	Test	Species	USEPA Source

NOAEC^1^	126 mg/kg-diet	Reproduction (USEPA2012g)	Northern Bobwhite	42055312
LC50	1536 mg/kg-diet	Dietary Toxicity Test (USEPA 2012f)	Northern Bobwhite	42055310
LD50	31 mg/kg-bwt (slope = 2.4)	Acute Toxicity Test (USEPA 2012e)	Japanese Quail (*Coturnix japonica*)	R2049931

1 Minimum NOAEC (pre-laying body weight), other endpoints from reproduction test provided in Supplementary Material.

**Table 3.** Pesticide application rates simulated in MCnest.

Pesticide	Application Rate (lbs Active Ingredient/Acre)

chlorpyrifos	1.0
imidacloprid	0.047
indoxacarb	0.11
λ-cyhalothrin	0.030
methomyl	0.45
permethrin	0.10

**Table 4.** MCnest predictions (fledglings/female/year and % reduction thereof compared to expectation of 1.04 in unexposed conditions) for a single application of each of six agricultural pesticides modeled using the terrestrial investigation model to estimate exposure.

Pesticide	Fledglings/Female/Year	% Reduction

chlorpyrifos	0.34	67
imidacloprid	0.86	18
methomyl	0.87	16
-λ-cyhalothrin	1.03	1
indoxacarb	1.03	0
permethrin	1.04	0

## Data Availability

All data used in this manuscript are publicly available in the supporting information to this manuscript or in published papers cited herein.
