# Supplementary material for: Using Pop-GUIDE to Assess the Applicability of MCnest for Relative Risk of Pesticides to Hummingbirds
Source: Ecologies (Basel). Author manuscript; Available in PMC 2024 Sep 4. (PMC11373374; doi:10.3390/ecologies4010013)
Supplement: Supplement1 [file NIHMS1954150-supplement-Supplement1.pdf]

ecologies
MDPI

Supplementary Material

Etterson et al. Using MCnest and Pop GUIDE to assess the relative risk of neonicotinoid pesticides to hummingbirds

Interest in pesticide residues in pollen and nectar residues from soil applications and seed treatments are an area of current research emphasis, with many recent publications measuring residues in a field setting. Additionally, over the past decade additional registration requirements for pesticides have included standardized residue test submitted to environmental agencies. Further publication and compilation of these studies is expected to enable significant improvements in algorithms that estimate residue concentrations. We publish current regulatory methods (from [45]) here but expect significant improvements in residue algorithms as compiled data are leveraged to evaluate candidate models.

Estimating pesticide concentrations in nectar and pollen from soil applications

Pesticide concentrations in pollen and nectar of crops growing in treated soil can be estimated using **Equation S1**, based on a model published by [96] and modified by [97]. This equation depends upon the K_{ow} and K_{oc} of a chemical as well as basic soil properties. Default values for soil properties include 0.01 for the fraction of organic carbon in soil (f_{oc}), a value of 1.5 g-dw/cm^3 for bulk density (ρ), and 0.2 cm^3/cm^3 is used for the soil water content (θ). Note that if K_{oc} is not available or appropriate for a chemical, the K_d can be substituted for the K_{oc} * f_{oc} term. The Transpiration Stream Concentration Factor (TSCF) is used to estimate pesticide transfer from the root system to aboveground plant tissues and can be calculated based on the Log K_{ow} of the assessed pesticide (**Equation S-2**).

Equation S1. $C_{pollen(t)}=C_{nectar(t)}=C_{soil(t)}*[10^{(0.95*LogKow-2.05)}+0.82]*TSCF*[\rho/(\theta+\rho*Koc*foc)]$

Equation S2. $TSCF = -0.0648 * (LogKow)^2 * 0.241 * LogKow + 0.5822$

Equation S3 gives the pesticide concentration in soil at time *t* by dividing the application rate (which is converted to kg a.i./ha by multiplying by 1.12) by the soil depth (d, in cm). A default depth of 15 cm (equivalent to 6 inches) is typically used [98] unless another depth can be justified. Degradation of the pesticide in soil is also accounted for using **Equation S3**, where the half-life value is the chemical-specific aerobic soil metabolism half-life ($t_{1/2(soil)}$, in days). This approach assumes no loss of the pesticide from soil via leaching, runoff, or volatilization.

Equation S3. $C_{soil(t)}=[(AR*1.12)/d]*e^{-kt}$

Estimating pesticide concentrations in nectar from seed treatments

For seed treatments, the European Plant Protection Organization [99] uses a screening value for pesticide concentration in pollen and nectar of treated crops of 1 µg a.i./g. This is considered a conservatively high value and more rigorous methods based on seed size and application rate would be a valuable area for future research. Empirical data is often used to refine conservative assumptions and reduce uncertainty associated with the above assumption. Appendix 1 and 2 of [45] provides conceptual

models of significant exposure concern and considerations for quantifying pesticide residues in pollen and nectar.

Parameter Documentation

Tables S1, S2, and S3 are provided to document models implemented in the main text.

Table S1. Parameter set for imidacloprid used for simulations to assess the relative risk of neonicotinoid pesticides to hummingbirds. Parameter = name of parameter in TIM/MCnest model, Value = value of parameter used for Ruby-throated Hummingbird simulations in TIM, Metadata = source and/or explanatory note about parameter.

Parameter	Value	Metadata
Model dietary exposure	yes	N/A
Model exposure through drinking from puddles	no	N/A
Model exposure through drinking from dew	no	N/A
Model exposure through dermal contact with foliage	no	N/A
Model exposure through dermal contact with spray	no	N/A
Model exposure off-field through spray drift	yes	N/A
Time of first application	8:00 AM	N/A
droplet spectrum	very fine to fine	N/A
Spray duration (min)	1.5	N/A
Crop height (m)	0.25	At time of application – consulted extension docs, see for example: https://www.ndsu.edu/agriculture/ag-hub/publications/soybean-growth-and-management-quick-guide<br>At V2, plants 6-8 inches tall<br>At R1, plants 12-14 inches tall<br>Average = 10 inches = 0.25m
Plant(crop) mass (kg/ha)	375	See procedure on p. 14 of TIM user guidance, some extension docs suggest plant biomass is about 2.5g at 50 d [99]. V1 occurs at around 25d and R1 occurs around 50d (SDSU extension service "Soybean Growth Stages"). MN extension recommends seeding rates of 125-150K seeds/acre (https://extension.umn.edu/soybean-planting/soybean-seeding-rates-minnesota). Assuming max seeding rate gives 375 kg/acre.

crop type	field	N/A
Fraction of edge habitat receiving spray drift	1	TIM default
Length of in field buffer (feet)	0	TIM default
fraction of organic carbon in soil	0.0128	MS soybean, from TIM guidance
soil bulk density (kg/L)	1.5	TIM default
Morning feeding start times: min and max	5:00 am, 5:00 am	"Morning" set to 5:00 am – 8:00 pm, with prop of feeding time in morning at 1. This generates the most uniform possible feeding schedule using TIM parameter choices.
Morning feeding end times: min and max	8:00 pm, 8:00 pm	N/A
afternoon feeding start times: min and max	n/a	Morning extended to full day for uniform feeding – see Morning Feeding Times
afternoon feeding end times: min and max	n/a	Morning extended to full day for uniform feeding – see Morning Feeding Times
Proportion of daily feeding taking place in morning: min and max	1	Morning extended to full day for uniform feeding – see Morning Feeding Times
Gorging factor	normal feeding	N/A
Contaminated fraction of food	1	N/A
Dislodgable foliar residue adjustment factor	0.62	TIM default, but see eqn. 6.5 in TIM User Guidance
Dermal adsorption fraction	1	N/A
avian acute inhalation LD50 (mg a.i.kg-bw)	Unavailable (0)	Not needed for this example, which did not include inhalation exposure
Chemical specific avian dermal LD50	Unavailable (0)	Not needed for this example, which did not include dermal exposure
Food matrix adjustment factor	1	TIM default
ratio of juvenile to adult toxicity	1	TIM default
Model exposure through vapor inhalation	no	N/A
Model exposure through spray inhalation	no	N/A

Number of applications	3	N/A
Application method (spray)	Aerial	N/A
Spray height	3 m	N/A
Rate of application #1 (lb a.i.A)	0.047	1/3 of labeled maximum application amount/season
Interval between app1 and 2 (days)	0	N/A
Rate of application #2 (lb a.i.A)	0	N/A
Interval between app2 and 3 (days)	0	N/A
Rate of application #3 (lb a.i.A)	0	N/A
Interval between app3 and 4 (days)	0	N/A
Rate of application #4 (lb a.i.A)	0	N/A
Interval between app 4 and 5 (days)	0	N/A
Rate of application #5 (lb a.i.A)	0	N/A
Food item half-lives (days)	35	TIM default
Pesticide half-life (days) in puddle	69	See guidance on p. 17 of TIM user manual http://npic.orst.edu/factsheets/archive/imidacloprid.html
K_{oc} (Lkg-oc)	292.5	http://npic.orst.edu/factsheets/archive/imidacloprid.html
K_{ow}	0.57	http://npic.orst.edu/factsheets/archive/imidacloprid.html
Henry's law constant (atm*m³/mol)	1.7E-10	http://npic.orst.edu/factsheets/archive/imidacloprid.html
solubility in water (mg a.i./L)	610	http://npic.orst.edu/factsheets/archive/imidacloprid.html
avian acute oral LD50 (mg a.i.kg/bw)	31	MRID = R2049931
Body weight of tested animals	130.4	MRID = R2049931
slope of avian oral LD50	2.4	MRID = R2049931
Mineau scaling factor	0.64<br>1.15	Default used for simulations, but fitted a unique curve for imidacloprid using six available LD50s, which gave an allometric slope of 0.64
Rat inhalation LD50 (mg a.i.kg/bw)	237	See rat inhalation data in: http://npic.orst.edu/factsheets/archive/imidacloprid.html However, not relevant with inhalation exposure turned off.
rat acute oral LD50 (mg a.i.kg/bw)	300	Female LD50 for rats from http://npic.orst.edu/factsheets/archive/imidacloprid.html

Hourly fraction of pesticide retained	0.719<br>0.974	Have RTHU-specific value from English et al. [18] of 0.719. USEPA/EFED sent a daily fraction retained value from Appendix 4-2 of the Imidacloprid BE. From this another value for hourly fraction retained was calculated to be 0.974. The latter was chosen as it was more conservative and was estimated via the same test protocol as for other chemicals.
Passerine vs. Non-passerine	Non-passerine	N/A
Altricial vs. precocial	Altricial	N/A
Body Weight	3.3g	[100]
Female body weight (g): mean, SD, min, max	3.3, 0.3, 2.6, 4.1	[100]
Male body weight (g): mean, SD, min, max	2.9, 0.2, 2.4, 3.7	[100]
feeding category: (insectivore, herbivore, granivore, omnivore)	insectivore	Nectarivore not possible in TIM, probably insensitive to this parameter
Fraction of each food item in diet (insects, seeds, fruit, grass, broadleaf)	0.5 Insects, 0.5 grass(nectar)	[50]
For juveniles: fraction of each food item in diet (insects, seeds, fruit, grass, broadleaf)	0.5 Insects, 0.5 grass(nectar)	[50]
Resident status (field vs. edge)	edge	Chosen to represent a bird that rarely forages on-field
Respiratory physiology adjustment factor	2.45	Smallest value available in TIM guidance, is 2.6 for a 10g bird, halving the bwt seems to result in step of about 0.1. Thus 2.45 was chosen to represent a 3.3g bird.
Frequency on field: mean, min, max	0, 0, 0.05	Chosen to represent a bird that rarely forages on-field
Fidelity factor	0	This parameter does not matter when FOF = 0
Passerine	no	N/A

Table S2. Full sensitivity results for ruby-throated hummingbird exposure to imidacloprid simulation. Columns labeled x0 and y0 represent the unperturbed parameter and response (fledglings/female/year), respectively. Columns labeled x1, x2, y1, and y2 are the backward and forward perturbed values of parameter and response, respectively. When x1=x0 or x2=x0, the parameter was already at the boundary condition and could not be further perturbed in that direction.

parameter	derivative	elasticity	x0	x1	x2	y0	y1	y2
Fraction of pesticide available from one hour to the next	-7.2011	-12.2882	0.974	0.9253	1	0.5708	0.5991	0.0612
Mineau	-4.60E-01	-0.9317	1.15	1.09	1.21	0.5675	0.5905	0.5376
Nestling period	-0.0235	-0.7364	18	17	19	0.5737	0.5942	0.5472
Application rate 1	-8.1656	-0.6724	0.047	0.0446	0.0494	0.5708	0.5903	0.5519
Incubation period	-0.0221	-0.6546	17	16	18	0.5737	0.5928	0.5486
m2	-9.4426	-0.4937	0.03	0.0285	0.0315	0.5737	0.5836	0.5553
Half-life - grass	-0.005	-0.3095	35	33.25	36.75	0.5708	0.5779	0.5603
m1	-5.4827	-0.2867	0.03	0.0285	0.0315	0.5737	0.5787	0.5622
Prop min	-0.1461	-0.2559	1	0.95	1	0.5708	0.5769	0.5696
Gorging factor	-0.1389	-0.2433	1	0.95	1.05	0.5708	0.5686	0.5547
Contaminated fraction - insects	-0.1362	-0.2386	1	0.95	1	0.5708	0.5797	0.5729
Half-life - Insects	-0.003	-0.1827	35	33.25	36.75	0.5708	0.5784	0.568
Prop max	-0.1042	-0.1825	1	0.95	1	0.5708	0.5764	0.5711
Contaminated fraction - grass	-0.0858	-0.1504	1	0.95	1	0.5708	0.5803	0.576
LD50 Bwt	-6.15E-04	-0.1413	130.4	123.88	136.92	0.5675	0.5719	0.5639
Egg-laying interval	-0.0376	-0.1311	2	1.9	2.1	0.5737	0.5764	0.5688
Adult diet - grass	-0.1356	-0.1188	0.5	0.475	0.525	0.5708	0.5795	0.5727
Spray height (m)	-0.0191	-0.1002	3	2.85	3.15	0.5708	0.5727	0.567
Residue half life	-0.0016	-0.0961	35	33.25	36.75	0.5675	0.5717	0.5663
Fractiion of edge habitat receiving spray drift	-0.0454	-0.0796	1	0.95	1	0.5708	0.5772	0.5749
FIR	-0.011	-0.0735	3.8	3.61	3.99	0.5675	0.5734	0.5692
Adult mortality	-12.6634	-0.053	0.0024	0.0523	0	0.5737	0.0218	0.6838
LC50 Bwt	-0.001	-0.0521	29.5	28.025	30.975	0.5675	0.5739	0.571
Contaminated fraction - broadleaf	-0.0278	-0.0488	1	0.95	1	0.5708	0.5723	0.5709
Rat inhalation LD50	-7.75E-05	-0.0322	237	225.15	248.85	0.5708	0.5723	0.5705
Am start min	-0.0035	-0.0307	5	4.75	5.25	0.5708	0.5731	0.5714
LC50	-1.12E-05	-0.0304	1536	1459	1613	0.5675	0.572	0.5703
Am end max	-4.93E-04	-0.0173	20	19	21	0.5708	0.5749	0.5739
Contaminated fraction - seeds	-0.0098	-0.0171	1	0.95	1	0.5708	0.5719	0.5714
Dislodgable foliar adjustment factor	-0.011	-0.012	0.62	0.589	0.651	0.5708	0.5705	0.5698
Field fidelity factor	-0.011	-0.0116	0.6	0.57	0.63	0.5708	0.5707	0.5701
Henry's law constant	-2.00E+07	-0.006	1.700E-10	1.615E-10	1.785E-10	0.5708	0.5683	0.5679
Ratio of juvenile to adult toxicity	-7.32E-04	-0.0013	1	0.95	1.05	0.5708	0.5718	0.5717
Pm start min	-8.99E-05	-7.87E-04	5	4.75	5.25	0.5708	0.5706	0.5706
Pm end max	-4.27E-06	-1.50E-04	20	19	21	0.5708	0.569	0.569
Half-life - fruits	1.06E-04	0.0065	35	33.25	36.75	0.5708	0.5676	0.568
Spray duration (min)	0.0027	0.0071	1.5	1.425	1.575	0.5708	0.5687	0.5691

Respiratory physiology adjustment factor	0.0018	0.0076	2.45	2.3275	2.5725	0.5708	0.5689	0.5693
Half-life - broadleaf	1.52E-04	0.0093	35	33.25	36.75	0.5708	0.5714	0.5719
Pesticide half life (puddle)	1.26E-04	0.0152	69	65.55	72.45	0.5708	0.5641	0.565
Juvenile diet - grass	0.0175	0.0153	0.5	0.475	0.525	0.5708	0.5718	0.5727
Fraction of organic carbon in soil	0.9499	0.025	0.015	0.0142	0.0158	0.5708	0.5713	0.5728
Soil bulk density	0.0095	0.0251	1.5	1.425	1.575	0.5708	0.5689	0.5703
Half-life - seeds	4.54E-04	0.0278	35	33.25	36.75	0.5708	0.5697	0.5713
Juvenile diet - insects	0.0359	0.0315	0.5	0.475	0.525	0.5708	0.5694	0.5712
Am start max	0.0041	0.0358	5	4.75	5.25	0.5708	0.5689	0.571
Dermal absorption factor	0.0229	0.0401	1	0.95	1	0.5708	0.5697	0.5709
Contaminated fraction - fruits	0.024	0.0421	1	0.95	1	0.5708	0.5712	0.5724
Kow	0.0443	0.0442	0.57	0.5415	0.5985	0.5708	0.5692	0.5718
Crop height (m)	0.1035	0.0453	0.25	0.2375	0.2625	0.5708	0.5675	0.5701
Pm start max	0.0052	0.0454	5	4.75	5.25	0.5708	0.5708	0.5733
Pm end min	0.0015	0.0519	20	19	21	0.5708	0.5712	0.5741
Clutch size	0.0154	0.0536	2	1.9	2.1	0.5737	0.5657	0.5688
LC50 Fraction	0.0609	0.0536	0.5	0.475	0.525	0.5675	0.5686	0.5716
Am end min	0.0016	0.055	20	19	21	0.5708	0.5728	0.5759
Crop mass (kg/ha)	1.19E-04	0.0781	375.0	356.3	393.8	0.5708	0.5676	0.5721
Solubility in water	8.62E-05	0.0921	610.0	579.5	640.5	0.5708	0.5677	0.573
Adult diet - insects	0.1159	0.1015	0.5	0.5	0.5	0.5708	0.5669	0.5727
Koc	2.23E-04	0.1144	292.5	277.9	307.1	0.5708	0.5691	0.5757
Rat acute oral LD50	2.32E-04	0.1221	300.0	285.0	315.0	0.5708	0.5661	0.5731
LD50	0.0035	0.1915	31.0	29.5	32.6	0.5675	0.5645	0.5754
Slope of avian oral LD50	0.0747	0.314	2.4	2.3	2.5	0.5708	0.5604	0.5783
Food matrix adjustment factor	0.2601	0.4074	1	0.95	1	0.6385	0.626	0.639

Table S3. Data used for estimating the Mineau scaling factor for imidacloprid. Body weights are taken from Dunning [100].

Species	LD50	M	F	Source
Japanese Quail[1]	23.7	93	96.6	MRID 44457401, 43310301
Common Quail	32.5	144	90	Ecotox #344
House Sparrow	41	28	27.4	MRID 42055309
Eared Dove	59	136	136	Ecotox #183555
Northern Bobwhite	152	178	178	MRID 42055308
Mallard	283	1246	1095	MRID 44059401

[1]Value used for modeling is the geometric mean of two estimates of the LD50 for Japanese quail, 17 and 33 mg/kg bodyweight respectively.

References:

18. English, S.G.; Sandoval-Herrera, N.I.; Bishop, C.A.; Cartwright, M.; Maisonneuve, F.; Elliott, J.E.; Welch, K.C. Neonicotinoid pesticides exert metabolic effects on avian pollinators. *Sci. Rep.* **2021**, *11*, 2914.

45. USEPA. Guidance for Assessing Pesticide Risks to Bees. Office of Pesticide Programs. 2014. Available online at: https://www.epa.gov/sites/default/files/2014-06/documents/pollinator_risk_assessment_guidance_06_19_14.pdf (accessed on 3 August 2022).

50. Weidensaul, S.; Robinson, T.R.; Sargent, R.R.; Sargent, M.B.; Zenzal, T.J. Ruby-throated Hummingbird (*Archilochus colubris*), version 1.0. In *Birds of the World*; Rodewald, P.G., Ed.; Cornell Lab of Ornithology: Ithaca, NY, USA, 2020.

96. Briggs, G.G.; Bromilow, R.H.; Evans, A.A.; Williams, M.; Relationships between lipophilicity and the distribution of non?ionised chemicals in barley shoots following uptake by the roots. Pesticide Science 1983, 14, 492-500.

97. Ryan, J.A.; Bell, R.M.; Davidson, J.M.; O'connor, G.A.; Plant uptake of non-ionic organic chemicals from soils. Chemosphere 1988, 17, 2299-2323.

98. USEPA Environmental Chemistry Methods Guidance. Office of Chemical Safety and Pollution Prevention 2012, Washington, DC, USA. Available online at: https://www.epa.gov/sites/default/files/2015-08/documents/ftt_env_chem_methods.pdf (ac-cessed on 20 August 2022).

99. Lenka, N.K.; Lenka, S.; Thakur, J.K.;, Elanchezhian, R.; Aher, S.B.; Simaiya, V.; Yashona, D.S.; Biswas, A.K.; Agrawal, P.K.; Patra, A.K.;. Interactive effect of elevated carbon dioxide and elevated temperature on growth and yield of soybean. Current Science 2017, 2305-2310.

100. Dunning, J.B. CRC Handbook of Avian Body Masses, 2nd Ed.; CRC Press: Boca Raton, FL, USA, 2008.
